# Cost and Affordability of Healthy, Equitable and Sustainable Diets in Low Socioeconomic Groups in Australia

**DOI:** 10.3390/nu13082900

**Published:** 2021-08-23

**Authors:** Meron Lewis, Sarah A. McNaughton, Lucie Rychetnik, Amanda J. Lee

**Affiliations:** 1 School of Public Health, Faculty of Medicine, The University of Queensland, Herston, QLD 4006, Australia; amanda.lee@uq.edu.au; 2 The Australian Prevention Partnership Centre, The Sax Institute, Glebe, NSW 2037, Australia; lucie.rychetnik@saxinstitute.org.au; 3 Institute for Physical Activity and Nutrition, School of Exercise and Nutrition Sciences, Deakin University, Geelong, VIC 3217, Australia; sarah.mcnaughton@deakin.edu.au; 4 School of Public Health, University of Sydney, Camperdown, NSW 2006, Australia

**Keywords:** diet cost, diet affordability, low socioeconomic, low income, healthy eating, dietary guidelines, Australia

## Abstract

Few Australians consume a healthy, equitable and more sustainable diet consistent with the Australian Dietary Guidelines (ADGs). Low socioeconomic groups (SEGs) suffer particularly poor diet-related health problems. However, granular information on dietary intakes and affordability of recommended diets was lacking for low SEGs. The Healthy Diets Australian Standardised Affordability and Pricing protocol was modified for low SEGs to align with relevant dietary intakes reported in the National Nutrition Survey 2011–2012(which included less healthy and more discretionary options than the broader population), household structures, food purchasing habits, and incomes. Cost and affordability of habitual and recommended diets of low SEGs were calculated using prices of ‘standard brands’ and ‘cheapest options’. With ‘standard brands’, recommended diets cost less than habitual diets, but were unaffordable for low SEGs. With ‘cheapest options’, both diets were more affordable, but recommended diets cost more than habitual diets for some low SEGs, potentially contributing to perceptions that healthy food is unaffordable. The study confirms the need for an equity lens to better target dietary guidelines for low SEGs. It also highlights urgent policy action is needed to help improve affordability of recommended diets.

## 1. Introduction

There is an urgent need for food system transformation to produce healthy, equitable and more environmentally sustainable diets for all people [1,2]. Poor diet is a leading contributor to the burden of disease in Australia [3,4]. Fewer than 4% of Australians consume a healthy, equitable and more sustainable diet consistent with the Australian Dietary Guidelines (ADGs) [5,6]. More than one-third of adults’ and more than 40% of children’s energy intake comes from “discretionary” food and drinks. These discretionary food and drinks are not needed for health and are high in saturated fat, added sugar, salt and/or alcohol [6]. Low socioeconomic groups (SEGs) suffer poorer diet-related health problems than the broader population, including higher rates of chronic disease such as diabetes, heart disease, and some cancers [7,8,9].

### 1.1. Key Considerations in Understanding Determinants of Inequitable Dietary Patterns

A recent systematic review of habitual dietary intake of low SEGs in Australia found that while total diet quality is generally lower in low SEGs compared to higher SEGs, findings were inconsistent across studies for all reported food groups and SEG measures due to variation between study metrics, definitions, dietary assessment methods, and granularity of analysis [10]. Most often, intakes of fruits and vegetables were used as markers of a healthy diet [10]. Quantitative intakes of ADG food groups by SEGs were reported rarely, and were not available readily from national survey data [5].

The inequities of healthy eating are complex, and strongly influenced by environmental, economic and social determinants [11]. The affordability of healthy food has been identified as a key leverage point in models of inequitable healthy eating, and is influenced by both household income and the cost of healthy food and drinks [12]. However, the relative cost of healthy and unhealthy food and drinks must be considered within the context of dietary patterns, rather than as individual components [13].

### 1.2. Food Habits and Incomes of Low SEGs

Low SEGs implement various food purchasing strategies to stretch the food budget. Low income households purchase a higher proportion of ‘own brand’ (also called generic brand, private label or home brand) products compared to higher income households [14]. The number of ‘own brand’ products in major Australian supermarket chains has been increasing [15] and purchasing those alternatives can deliver large cost savings [16]. The number of ‘budget’ supermarkets in Australia has also been increasing, providing a popular source of low cost groceries [15]. Additionally, household expenditure surveys have shown that low income households spend less on eating out and takeaway foods than higher income households: purchase of convenience foods from supermarkets by low SEGs approximates that of foods consumed away from home by high SEGs [17].

Two-thirds (65%) of households in the lowest household income quintile in Australia report government pensions and allowances as their main source of income [18]. Assessment of healthy food affordability for households receiving a low income or reliant on payments such as unemployment benefits or aged pension will highlight the inequities faced by these vulnerable groups.

### 1.3. The Healthy Diets ASAP Protocol

In Australia, there was an urgent need for comparable food cost and affordability data to inform fiscal policy from a health perspective. A previous review identified 11 different methods that had been used in Australia and there was a lack of comparability across all metrics, with approaches rarely fully aligned with recommendations of the ADGs, and only one attempted comparison with the cost of a typical diet. [19]. The Healthy Diets Australian Standardised Affordability and Pricing (ASAP) method protocol was developed to compare the cost, cost differential and affordability of habitual (current, typically unhealthy) and recommended (healthy, equitable and more sustainable) omnivorous diets for the mean population in Australia [20].

This standardised approach includes a five-part protocol:

1. Habitual (current, unhealthy) and recommended (healthy, equitable, more sustainable) diet pricing tools, including foods commonly consumed by the Australian population, for reference households
2. Store location and sampling
3. Food and drink price data collection
4. Calculation of median household income, and low-income household income (minimum wage plus welfare payments) for the reference households
5. Analysis and reporting

The types and amounts of the food and drinks in the habitual diet pricing tool were sourced from mean dietary intakes reported by reference household members in the most recent national survey [21]. The recommended diet pricing tool includes those healthy food and drinks in the habitual diet pricing tool, in higher quantities reflecting ADG recommendations. Recommended diets in Australia promote health and wellbeing, are equitable [6] and are more environmentally sustainable with modelling reporting generation of 25% less greenhouse gas emissions, than habitual diets [22].

Implementation of the protocol has shown that, under present fiscal policy settings in Australia where basic healthy food and drinks do not incur the 10% Goods and Service Tax (GST), healthy diets are between 16–24% less expensive than habitual diets but are still unaffordable for many Australians. [23,24,25]

Consultations with academic, government and non-government organisations to inform development of the Healthy Diets ASAP protocol for the mean population noted requests to also develop methods specific to low socioeconomic and other groups [20]. The protocol was modified subsequently to reflect dietary intakes and circumstances of Aboriginal and Torres Strait Islander peoples, which resulted in a more sensitive tool to describe the cost and affordability of habitual and recommended diets in these population groups [23].

Modification of the Healthy Diets ASAP protocol for low SEGs would provide more granular evidence to better target dietary guidelines for low SEGs and inform policies and practices to help low SEGs purchase and consume healthy diets and improve diet-related health. Improved health outcomes for low SEGs may result in reduced health costs, improved workforce and social participation, improved education outcomes for children, and reduced social inequality [9].

The aim of this study was twofold: (i) to modify the relevant components of the original Healthy Diets ASAP protocol to accommodate habitual dietary intakes, household structures, food purchasing habits, and income sources and amounts, of low SEGs in Australia; and (ii) to test the low SEG Healthy Diets ASAP protocol to assess the cost, cost differential and affordability of habitual and recommended diets for low SEG households in Australia.

## 2. Materials and Methods

### 2.1. Development of the Healthy Diets ASAP Protocol for Low SEGs in Australia

As relevant quantitative habitual dietary intake data were not available (as noted above) reported dietary intakes of individuals in low SEGs from the most recent Australian Health Survey National Nutrition and Physical Activity Survey (AHS NNPAS) in 2011–2012 were used to modify the habitual diet pricing tool [26]. The recommended diet pricing tool did not require modification, as healthy, equitable and more sustainable dietary recommendations are similar for all Australians [6]. The modified pricing tools were tested iteratively and results informed development of the low SEG protocols. The methods and results for the tools and testing are reported separately.

#### 2.1.1. Selection of SEG Measure

Household income was used as the indicator of SEG in this study, as this metric reflects household resources to purchase food, and is available for all persons surveyed in the AHS NNPAS. When examining categories of income, low sample numbers within subcategories in the AHS NNPAS (see Table S1) dictated use of income quintiles, rather than the deciles reported publicly [21]. Low SEGs were defined as those in the lowest income quintile.

#### 2.1.2. Selection of Low SEG Reference Households

Three common household compositions among low SEGs in Australia comparable with households in the Healthy Diets ASAP protocol [18] were included. Additionally, to account for low sample numbers within age subcategories in the AHS NNPAS (Table S1), the original included age range for the children was expanded. The low SEG reference households were:

- Household A: Two adults (female 31–50 years, male 31–50 years) and two children (boy 14–18 years, child 4–8 years)
- Household B: One adult (female 31–50 years) and two children (boy 14–18 years, child 4–8 years)
- Household C: Older, retired couple (female 70+ years, male 70+ years)

#### 2.1.3. Modification of the Habitual Diet Pricing Tool for Low SEGs

Dietary intake was collected in the AHS NNPAS [21] using 24-h dietary recall. The Confidential Unit Record Files of the AHS NNPAS were assessed and analysed to determine mean intake of food and drinks of members of the reference households, by age, gender, and household income quintile. The mean intakes of all food and drinks for the lowest income quintile for each reference household (sum of mean intakes of household members) were then mapped to the 75 representative food and drinks of the habitual diet pricing tool (Table S2). The low SEG habitual diet pricing tool was analysed for energy content using the FoodWorks 9th Edition computer program [27].

#### 2.1.4. Modification of the Store Location and Sampling Methods for Low SEGs

For the store location and sampling methods, ‘budget’ supermarkets (e.g., ALDI^®^ stores), were included, in addition to the major supermarkets and other food outlets of the original Healthy Diets ASAP protocol.

#### 2.1.5. Modification of the Price Collection Methods for Low SEGs

In the low SEG price collection methods, prices were collected for the most commonly purchased brands in Australia as a whole (‘standard brands’), as per the original Healthy Diets ASAP protocol, and prices were also collected for the ‘cheapest option’ available, usually an ‘own brand’ product. As non-packaged produce such as fruit, vegetables, and meats are not branded, these items were selected by type alone and the same prices were included in both ‘standard brands’ and ‘cheapest option’ price collections.

#### 2.1.6. Household Income Calculations for Low SEGs

The low-minimum disposable household income of the original Healthy Diets ASAP protocol was calculated including minimum wage rates [28], tax payable [29] and any applicable welfare payments [30], and this was retained for the low SEG protocol. A welfare dependent household income, calculated to include only welfare payments such as unemployment benefits, was added to the low SEG protocol.

#### 2.1.7. Modification of the Analysis and Reporting Methods for Low SEGs

The analysis and reporting component of the low SEG Healthy Diets ASAP protocol was modified to include additional calculation of costs and affordability using the ‘cheapest option’ price collection. Costs for habitual and recommended diets were reported as total cost and cost of each ADG food group or food group component, for the ‘standard brands’ and ‘cheapest option’ price collections.

The cost of ‘healthy’ food in the habitual diet was the sum of costs of those foods and drinks listed in the recommended diet together with artificially sweetened drinks. The cost of ‘discretionary’ food and drinks in the habitual diet was the sum of costs of those food and drinks not included in the recommended diet.

Diet costs were deemed unaffordable if they were more than 30% of household income [31]. If diet costs were more than 25% of disposable household income, the household was considered to be in food stress [32,33].

### 2.2. Testing the Healthy Diets ASAP Protocol for Low SEGs in Australia

#### 2.2.1. Food and Drink Price Collection

To test the low SEG protocol, food and drink prices were collected in June 2020 from one conveniently sampled Statistical Area 2 (SA2) in Brisbane, Queensland, Australia, using the Healthy Diets ASAP web-based data collection portal [34]. Due to restrictions related to the SARS-CoV-2 pandemic at the time, the majority of food and drink prices were collected online from two major supermarket and liquor store chains. Food and drink prices at a budget supermarket (ALDI^®^) and prices from other stores included in the original protocol (independent bakery, fish and chip store, burger restaurant chain store, and pizza chain store) were collected in-store as these were unavailable online. Prices collected included both ‘standard brands’ and ‘cheapest option’ packaged products.

#### 2.2.2. Data Analysis

Data analysis was conducted using algorithms with the following steps: item prices and sizes were entered into the Healthy Diets ASAP web-based data collection portal; prices were converted to price per gram or millilitre, adjusted by an edible portion factor (to account for cooking or inedible parts), and then multiplied by the amount consumed by the reference household per fortnight as per the diet pricing tools. Individual food and drink prices were then summed to provide a total cost for each ADG food group or food group component, and the total diet cost per household per fortnight. Diet costs were calculated based on the ‘standard brands’ price collection (from major supermarkets) and the ‘cheapest option’ price collection (from major supermarkets and the budget supermarket) for each of the three low SEG households.

Household income was calculated in two different ways where relevant for each of the three low SEG households: (i) for those on a welfare only income, and (ii) for those working age adults on a minimum wage-based income. Detailed calculations of the household incomes are included in Table S3. Affordability of both habitual and recommended diets was calculated for each household and relevant income levels.

## 3. Results

### 3.1. The Low SEG Healthy Diets ASAP Protocol

The components of the original Healthy Diets ASAP protocol and the low SEG Healthy Diets ASAP protocol are shown in Table 1, with further details below.

#### 3.1.1. The Low SEG Habitual Diet Pricing Tools

Details of the low SEG habitual diet pricing tool for Household A (two adults, two children) are shown in Table 2, together with the composition of the original Healthy Diets ASAP habitual diet pricing tool. Equivalent data for Households B (one adult, two children) and C (older, retired couple) are presented in Table S4A,B.

The habitual diets of low SEG Households A, B and C provided 97%, 98% and 99% respectively of the energy content of habitual diets for the mean Australian population (that is, as described in the original Healthy Diets ASAP protocol) (Table 2 and Table S4A,B), and 99%, 99% and 98%, respectively, of the total energy intake reported by members of these households in the AHS NNPAS [21]. The energy content of the habitual diets of low SEG Households A, B and C provided 99%, 99% and 95% respectively of the energy content of the recommended diets for the same households (Table 2 and Table S4A,B).

Overall, energy derived from healthy food and drinks in the habitual diets of low SEGs was 10%, 11% and 3% lower, respectively, for Households A, B, and C than habitual diets of the mean population (Table 2). Energy derived from discretionary food and drinks in the habitual diets of low SEGs was 2% higher, 6% higher and no different, respectively for Households A, B, and C, than habitual diets of the mean population.

Compared to habitual diets of the mean population, habitual diets of low SEGs included, by weight, for Households A, B and C respectively: 14%, 14% and 8% less fruit; 6%, 13% and 4% less vegetables and legumes; 11% less, 14% less and 10% more grain (cereal) foods, and 19%, 17% and 6% less lean meats, poultry, fish, eggs, nuts, and seeds (“lean meats etc.”). However, habitual diets of low SEGs included, by weight, for Households A, B and C respectively: 17%, 21% and 5% more takeaway foods; 36% more, 58% more and 2% less sugar sweetened beverages (SSBs); and 41%, 60% and 27% less artificially sweetened soft drinks than habitual diets of the mean population (Table 2 and Table S4A,B).

#### 3.1.2. Price Collection

When the food price collection methods were modified to accommodate ‘cheapest option’ items in the low SEG protocol, the revised wording for data collection was: “When collecting the ‘cheapest option’ prices, the price of the cheapest equivalent product (selected from all brands including ‘own brands’) in the specified size is collected. For the items pie, pizza, and chips, usually sourced from other stores, the price of a frozen equivalent item from the supermarket is collected, selecting the cheapest option from all brands, including ‘own brands’, in the specified size. The takeaway burger item should be priced from the burger restaurant as per the original protocol. If the specified size is not available, choose the nearest larger size. If a larger size is not available, choose the nearest smaller size.” Testing the low SEG protocol showed that including ‘cheapest option’ products resulted in marked cost reductions compared to the ‘standard brands’ products. Of the 60 packaged foods priced in supermarkets and discount supermarkets, 52 (87%) were an ‘own brand’ equivalent, five (8%) were a ‘cheapest brand’ equivalent, and three (5%) were ‘standard brands’.

#### 3.1.3. Modifications of Sources and Amounts of Household Income

The welfare dependent household income and low-minimum disposable household income amounts for each reference household, and the assumptions made in their calculation for the low SEG protocol, are shown in Table S5.

### 3.2. Testing of the Low SEG Healthy Diets ASAP Protocol

**Figure 1:**
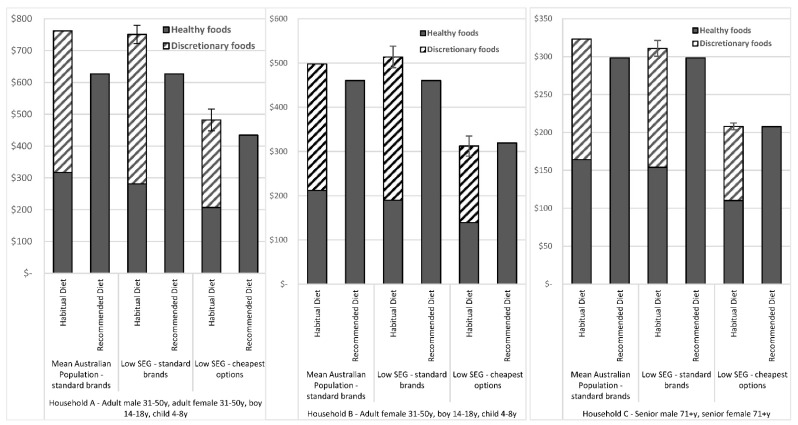
Costs of habitual and recommended diets for mean population and low SEG reference households (using ‘standard brands’ and ‘cheapest options’) per fortnight. Error bars reflect standard errors. y = years.

The costs of habitual and recommended diets for the three reference households, calculated by application of the low SEG protocol for both ‘standard brands’ and ‘cheapest options’, and the costs calculated by application of the original Healthy Diets ASAP protocol (mean population intakes), are shown in Figure 1, and detailed below.

Detailed costs of the component food groups of habitual and recommended diets, for the mean population and low SEG reference households per fortnight, are shown in Table S6A–C.

#### 3.2.1. Comparison of Habitual and Recommended Diet Costs Determined by the Low SEG Protocol and the Original Healthy Diets ASAP Protocol Using ‘Standard Brands’

##### Comparison of Total Costs of Diets Calculated by the Low SEG Protocol and the Original Healthy Diets ASAP Protocol

When ‘standard brands’ were priced, the total costs of the habitual diets of low SEGs were 1% lower ($11 per fortnight) for Household A, 3% higher ($16 per fortnight) for Household B, and 4% lower ($13 per fortnight) for Household C, than habitual diet costs for the mean population (Figure 1). As the recommended diet pricing tool was the same, the cost of the recommended diet for low SEGs and the mean population was also the same.

##### Comparison of Diet Costs of Food Groups and Food Group Components Calculated by the Low SEG Protocol and the Original Healthy Diets ASAP Protocol

When ‘standard brands’ were priced, the healthy food and drink costs of the habitual diets of low SEG were 11% ($36 per fortnight) lower for Household A, 10% ($22 per fortnight) lower for Household B and 6% ($10 per fortnight) lower for Household C, than healthy food and drink costs for the mean population. The discretionary food and drink costs of the habitual diets of low SEG were 6% higher ($25 per fortnight) for Household A, 13% higher ($38 per fortnight) for Household B, and 1% lower ($2 per fortnight) for Household C than the discretionary food and drink costs of the mean population (Figure 1). Costs in habitual diets of low SEGs for fruit, vegetables and legumes; grain (cereal) foods; lean meats and poultry, fish, eggs, nuts and seeds; and artificially sweetened soft drinks, were lower, and costs for: takeaway foods and SSBs were higher than costs for the mean population, in all low SEG households. (Table S6A–C).

##### Comparison of Habitual Diet and Recommended Diet Costs

When ‘standard brands’ were priced, the cost of the recommended diet was less expensive than the habitual diets of low SEGs, by 17% ($124 per fortnight) for Household A, 10% ($53 per fortnight) for Household B, and 4% ($13 per fortnight) for Household C (Figure 1).

##### Proportion of Total Habitual Diet Costs Spent on Discretionary Food and Drinks

When ‘standard brands’ were priced, the proportion of the food budget of low SEGs spent on discretionary items was 63% ($470 per fortnight) for Household A, 63% ($324 per fortnight) for Household B, and 50% ($157 per fortnight) Household C (Figure 1).

#### 3.2.2. Habitual Diet Cost Differences between ‘Standard Brands’ and ‘Cheapest Options’

When ‘cheapest options’ were priced instead of ‘standard brands’, the cost of habitual diets of low SEGs reduced by around 36%, and the cost of the recommended diets reduced by around 31%. (Figure 1). When ‘cheapest options’ were priced instead of ‘standard brands’, the cost of the recommended diet was 10% less ($48 per fortnight) for Household A, 2% more ($7 per fortnight) for Household B, and equal cost to the habitual diet for Household C.

#### 3.2.3. Affordability of Habitual and Recommended Diets Using ‘Standard Brands’ and ‘Cheapest Options’

**Figure 2:**
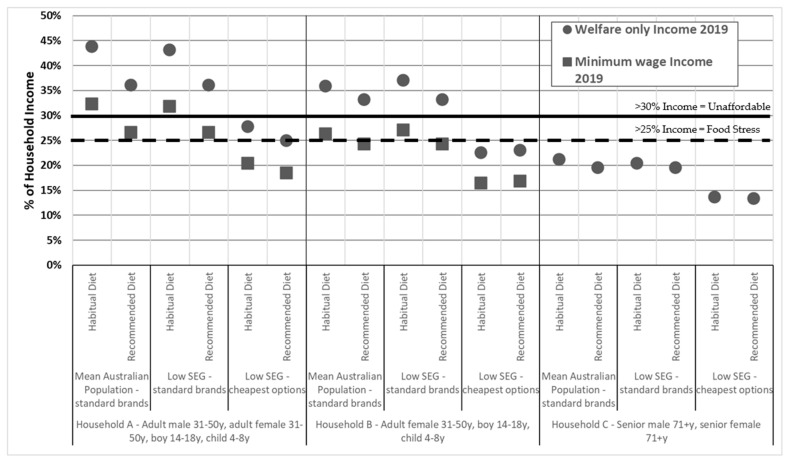
Affordability of habitual and recommended diets for mean population and low SEG reference households receiving welfare only and minimum wage incomes (using ‘standard brands’ and ‘cheapest options’). y = years.

The affordability of habitual and recommended diets for mean population and low SEG reference households (using ‘standard brands’ and ‘cheapest options’ prices) are shown in Figure 2. Affordability of the diets are shown for Households A and B at two calculated household incomes: a low-minimum disposable and a welfare only income, and for Household C, at a calculated welfare only income (as both members of this household are retired and not receiving employment income).

##### Affordability of Habitual and Recommended Diets for Household A (Two Adults, Two Children)

For Household A receiving the low-minimum disposable income, when ‘standard brands’ were priced, habitual diets of low SEGs cost 32% of household income. Recommended diets cost 27% of household income. When purchasing ‘cheapest options’ habitual diets of low SEGs and recommended diets required 20% and 18%, respectively of the low-minimum household income (Figure 2).

For Household A receiving a welfare only income, when ‘standard brands’ were priced, habitual diets of low SEGs cost 43% of household income (Figure 2). Recommended diets required 36% of the welfare household income. If the household purchased ‘cheapest options’, habitual and recommended diets required 28% and 25%, respectively of the welfare household income (Figure 2).

##### Affordability of Habitual and Recommended Diets for Household B (One Adult, Two Children)

For Household B receiving the low-minimum disposable income, when ‘standard brands’ were priced, habitual diets of low SEGs cost 27% of household income. Recommended diets cost 24% of household income. When purchasing ‘cheapest options’, habitual and recommended diets required 16% and 17%, respectively, of the low-minimum disposable income (Figure 2).

For Household B receiving a welfare only income, when ‘standard brands’ were priced, habitual diets of low SEGs cost 37% of the welfare household income. Recommended diets required 33% of the welfare household income. When ‘cheapest options’ were purchased, both the habitual diet and the recommended diet required 23% of the welfare household income (Figure 2).

##### Affordability of Habitual and Recommended Diets for Household C (Older, Retired Couple)

For Household C on a welfare only income, when ‘standard brands’ were priced, habitual diets of low SEGs cost 20% of the welfare household income. Recommended diets also required 20% of the welfare household income. When ‘cheapest options’ were purchased, habitual and recommended diets required 14% and 13%, respectively, of the welfare household income (Figure 2).

## 4. Discussion

### 4.1. Summary of Findings

Development and testing of the low SEG Healthy Diets ASAP protocol showed that overall energy content and cost of habitual diets for each reference low SEG household was similar to that of the corresponding mean population reference households (assessed by the original Healthy Diets ASAP protocol). However, in the habitual diets of low SEGs a higher proportion of energy, and cost, was derived from discretionary food and drinks, particularly SSBs and takeaway foods, with a corresponding decrease in energy and cost derived from healthy food and drinks and artificially sweetened beverages.

The habitual diet was more expensive than the recommended diet for all three low SEG reference households when ‘standard brands’ were purchased. However, when the ‘cheapest options’ were purchased instead of ‘standard brands’, habitual diets of low SEGs cost the same as recommended diets for Household C (older, retired couple), became less expensive than recommended diets for Household B (one adult, two children), but remained more expensive than recommended diets for Household A (two adults, two children).

For recommended diets to be affordable (<30% of disposable income) for Households A and B receiving a minimum wage income, it was necessary to employ strategies such as purchasing ‘cheapest option’ products. When Households A and B were reliant upon welfare benefits, affording recommended diets would be even more challenging. Recommended diets for Household C receiving a welfare only income would be more affordable than the other households on a welfare only income.

### 4.2. Differences between Habitual Diet of Low SEGs and the Mean Population

When ‘standard brands’ were priced, the habitual diet of low SEGs was more expensive than recommended diets, consistent with the findings of previous applications of the original Healthy Diets ASAP protocol [23,25,35]. This is partially due to exemption of basic, healthy food and drinks from GST in Australia [36].

Total diet costs and affordability of habitual diets priced using ‘standard brands’ were similar for low SEGs and the mean Australian population. However, analysis of each food group showed lower cost contributions from healthy food and drinks, and higher cost contributions from discretionary food and drinks in the habitual diets of low SEGs compared to those of the mean population. These cost differences reflect dietary intake differences between low SEGs and the mean Australian population, as captured in the respective diet pricing tools. Such variation may relate to differing perceptions that healthy foods are too expensive, lack of food preparation time and resources among different SEGs, and higher promotion of unhealthy foods in the food environment in low SEG areas [2,11,37,38]. Additionally, many complex social barriers affect access to resources, which in turn influence food choice in low SEGs [10,39].

The costs of habitual diets in all low SEG reference households (exemplified here by data for Household A) included a higher proportion spent on SSBs (5.5% of total habitual diets in low SEG), and lower proportion spent on artificially sweetened beverages (0.5% of total habitual diets in low SEG), in comparison to the mean population (4.0% and 0.8%, respectively, of total habitual diets in the mean population). These differences may be one reason that, although potentially regressive (i.e., having greater impact on low SEGs), other studies have postulated greater health benefits of a tax on SSBs to low SEGs than the rest of the population [40].

Similarly, costs of habitual diets of all low SEG reference households included a higher proportion from takeaway foods compared to the mean population. In contrast, household expenditure surveys show that low SEGs spend less on ‘meals out and fast foods’ than higher SEGs [17]. However, expenditure surveys solely reflect the purchase location, rather than the nutritional quality of food being purchased. Our results correlate with other studies that suggest that when low SEGs consume food prepared outside the home, they tend to purchase ‘fast food’ rather than healthier meals, such as in restaurants [41,42].

### 4.3. Choice of ‘Cheapest Options’ as a Coping Strategy to Stretch the Budget

Purchase of ‘cheapest options’ instead of ‘standard brands’ resulted in cost savings of 31% for recommended diets, and 36% for habitual diets. These differences arose as more packaged products are included in the latter than the former. The cost differential between habitual and recommended diets reduced to zero for Household C, and habitual diets became less expensive than recommended diets for Household B. For Household A, habitual diets were still more expensive than recommended diets when ‘cheapest options’ were purchased instead of ‘standard brands’, but the cost differential was smaller. This reduction and reversal in the cost differential may help explain the common perception that healthy food is more expensive than unhealthy food [11], and may be a driver for the consumption of unhealthy packaged foods over fresh healthy foods in low SEGs.

Other coping strategies that may be used by low SEG households to stretch their food budget include taking advantage of price promotions. However, a previous study found price promotions may save only a small (3%) proportion of cost for both habitual and recommended diets [14]. Discounted food and drinks tend to be less healthy than other products, and thus this can reduce the quality of habitual diets [43]. Therefore, households that adjust their shopping habits by stockpiling price promoted products to consume later may be able to save in the medium term, but this practice can also lead to increased consumption [44].

### 4.4. Affordability of Diets

Recommended diets were unaffordable for Households A and B, when receiving welfare benefits, but were affordable for Household C receiving the aged pension. The aged pension is indexed to average wages in Australia, whereas unemployment benefits are indexed to inflation. The aged pension has increased at a greater rate than unemployment benefits, which did not increase in real terms from 2009 to 2020 [45].

### 4.5. Strengths of the Low SEG Healthy Diets ASAP Protocol

Face validity of the low SEG habitual diet pricing tool was supported, as the energy content of the low SEG habitual diet for each reference household was within 2% of the energy content of corresponding reported energy intakes in the AHS NNPAS [21].

#### 4.5.1. Selection of SEG Measure

For the purposes of this study, household income was selected as the measure of low SEGs as it reflected household resources for food purchases, even if a recent lifestyle change had occurred, such as job loss or family separation. Many households comprised of older people may report a low income, despite having access to retirement savings and superannuation for daily expenditure. However, household income was preferred over household asset levels to indicate the SEG of older households, as such assets are not usually available to spend on daily expenses. Other SEG measures used in dietary intake studies in Australia included education, occupation, disadvantage level of the residential area, and/or combinations thereof, although household income was most commonly used [10]. Some previous studies found differences in SEG gradients of dietary intake using different measures of SEG [46,47,48], however measures such as education and occupation are not available in the AHS NNPAS for all reference household members [26].

#### 4.5.2. Selection of Low SEG Households

By including three types of low SEG households instead of just one, we have increased the range of relevant tools available to future users of the low SEG Healthy Diets ASAP. This study was also able to demonstrate how the cost, cost differential, and affordability of diets varied for different, common, low SEG household composition types.

### 4.6. Limitations

There are inherent limitations of the original Healthy Diets ASAP protocol that also apply to the low SEG protocol [20]. These include underlying assumptions: that food is equitably shared with all household members; that there is minimal food wastage; and that food is not acquired through home production. Measurement of dietary intake by 24-h recall (as in the AHS NNPAS) is known to be biased due to social desirability, particularly among low SEGs [26,49]. As with the original protocol, no adjustments have been made to account for the likely under-reporting of overall food intake and over-reporting of healthier foods. Hence, the findings of this study present a ‘best-case’ scenario.

As with the original Healthy Diets ASAP protocol, due to the sampling methods of the AHS NNPAS, it was not possible to analyse dietary intakes of actual family groups, as only one adult, or one adult and one child, were selected from households included in the national dietary survey [26]. This may have impacted particularly the low SEG habitual diet pricing tools as, for example, dietary intakes of children in single parent households may differ from those of children in two parent households. Further, low sample size numbers within subcategories of some age/gender/income groups in the AHS NNPAS 2011–2012 [21] affected the reliability of mean dietary intakes calculated for teenage boys (included in Households A and B), and older adults (included in Household C). However, despite these limitations, the AHS NNPAS 2011–2012 was the most detailed, recent, national source of population dietary intake data for this study [10].

For monitoring and surveillance purposes, it is essential that a standardised tool is used to collect current food and drink prices. The AHS NNPAS (2011–2013) data used in the development of the standardised habitual diet pricing tool are the most recent available in Australia, but are now 10 years old, and dietary intake patterns may have altered over those years. While few changes were noted between the 1995 National Nutrition Survey and the AHS NNPAS 16 years later [50], recent food environment changes (such as the rise in online food delivery options) may have influenced current dietary intakes. Additionally, the effects of the SARS-CoV-2 pandemic on employment (affecting income) and movement restrictions (affecting locations available for food shopping) may have been particularly challenging for low SEGs.

Testing the low SEG protocol used prices from a major city location. Regional and/or remote areas are likely to experience higher food prices [51] and a relative lack of ‘own brand’ products and budget supermarkets [52]. Therefore, diet cost and affordability results in this study reflect a ‘best case scenario’ for low SEGs.

Incomes were calculated at 2019 rates to avoid enumerating complex economic support supplements instituted by the Australian Government in response to the SARS-CoV-2 pandemic. The consumer price index for food in Queensland increased by 3.5% between June 2019 and June 2020, and thus diet affordability may be slightly underestimated [53].

The low SEG Healthy Diets ASAP protocol targets those in the lowest quintile of household income. However, some of these low SEG households experience particular challenges, such as very low incomes (due to ineligibility for welfare benefits), homelessness or unstable housing, limited access to food stores, and/or particular cultural food requirements. The low SEG Healthy Diets ASAP protocol does not specifically capture dietary intakes or incomes of these extremely vulnerable groups.

Additional coping strategies that may be used by low SEGs to stretch the food budget, such as shopping at market stalls or culturally specific stores, bulk purchasing, and/or accessing food banks, charitable donations, subsidised meals or food provided by family or friends, have not been included in this study [54,55,56].

### 4.7. Policy Implications

Our findings reflect reported dietary intake differences between low SEGs and the broader population, which have not been quantified previously across all ADG food groups [10]. This study confirms the need for an equity lens to better target the ADGs to low SEGs in Australia [6].

One measure to improve affordability of healthy, equitable and more sustainable diets is to increase household income. In the early months of the SARS-CoV-2 pandemic in 2020 the Australian Government implemented a number of economic stimulus measures to combat the sudden increase in unemployment [57]. This resulted in an increased income for many welfare dependent households and thus improved affordability of recommended diets [58]. A national survey found 83% of welfare dependent families reported eating healthier and more regularly compared to pre-pandemic times [59]. While these economic measures were only of short duration, this tangible example demonstrated the beneficial impact of increasing welfare support to adequate levels.

The results of the study also suggest that there is an opportunity to encourage purchase and consumption of recommended diets by making unhealthy foods relatively more expensive than healthy foods. Provision of vouchers for healthy food, such as the Special Supplemental Nutrition Program for Women, Infants and Children (WIC) in the USA, have been shown to increase consumption of healthy foods [60]. Promotion and discounting of healthy, rather than unhealthy, foods and beverages may also encourage their purchase [43]. In Australia, in remote Aboriginal and Torres Strait Islander communities, a study restricting promotion of unhealthy foods decreased their consumption [61]. Further research testing the impact of providing discounts for healthy foods for families with young children is currently underway (Ferguson et al., unpublished results), contributing to important evidence of potential policy changes to address inequities in dietary patterns.

Increasing taxation of unhealthy foods has also been suggested [25,62]. Increasing the GST rate on unhealthy foods and retaining the current exemption of GST on basic, healthy foods, increases the relative cost of unhealthy foods. Modelling has shown that increasing the rate of GST to 20% on unhealthy foods would make recommended diets 9% more affordable than habitual diets and raise revenue that could be used for health promotion programs [24].

By creating more supportive fiscal environments, such regulatory policy measures would help address the dietary inequities faced by low SEGs [39,63]. Reduction of economic barriers to healthy eating would also provide greater opportunity for low SEG households to benefit from nutrition education and food literacy programs [39,63].

## 5. Conclusions

Development of the low SEG Healthy Diets ASAP protocol enables calculation of habitual and recommended diet costs and affordability that assimilate the habitual dietary intakes, household structures, food purchasing habits, and income sources and amounts of low SEGs in Australia. The low SEG habitual diet pricing tool incorporates differences in dietary intake between low SEGs and the mean Australian population including lower quantities of healthy food and drinks and higher quantities of key discretionary food and drinks, particularly takeaway foods and SSBs.

Testing the low SEG protocol showed affordability of both diets improved when ‘cheapest options’ were purchased, but that the cost differential between habitual and recommended diets decreased. The finding that for some low SEG households recommended diets became more expensive than habitual low SEG diets could partly explain commonly-held perceptions that healthy food is unaffordable [11].

Policy action is necessary to increase affordability of recommended diets for low SEGs by reducing healthy food and drink costs and ensuring all household incomes are sufficient. This should include measures aimed at increasing the differential between costs of habitual and recommended diets, and at supporting and encouraging low SEGs to purchase and consume healthy diets.

Further application of the low SEG Healthy Diets ASAP protocol will provide additional data to inform policy and practice change. Improving diet-related health will lead to reduced health costs, improved workforce and social participation, improved education outcomes for children, and reduced social inequality, thus benefiting all Australians.

## Figures and Tables

**Table 1:** Components of the original Healthy Diets ASAP protocol and the Low SEG Healthy Diets ASAP protocol.

Protocol Component	Original Healthy Diets ASAP Protocol	Low SEG Healthy Diets ASAP Protocol **
Reference households	Household A: Adult male (31–50 years), Adult female (31–50 years), Boy (14 years), Girl (8 years) Household B: Adult female (31–50 years), Boy (14 years), Girl (8 years) Household C: Senior male (71+ years), Senior female (71+ years)	Household A: Adult male (31–50 years), Adult female (31–50 years), Boy (***14–18 years***), Child (***4–8 years***) Household B: Adult female (31–50 years), Boy (***14–18 years***), Child (***4–8 years***) Household C: Senior male (71+ years), Senior female (71+ years)
Habitual (current, unhealthy) diet pricing tool	Mean dietary intakes reported by specific age and gender individuals in AHS NNPAS, abridged and combined to provide household diet per fortnight	Mean dietary intakes reported by specific age and gender individuals ***of lowest household income quintile*** in AHS NNPAS, abridged and combined to provide household diet per fortnight
Recommended (healthy, equitable, sustainable) diet pricing tool	Healthy food and drinks included in Habitual diet pricing tool in amounts reflecting ADG.	Healthy food and drinks included in Habitual diet pricing tool in amounts reflecting ADG.
Store location and sampling methods	Major supermarkets and other food outlets	Major supermarkets, ***budget supermarkets*** and other food outlets
Food and drink price data collection methods	Prices collected of non-packaged items and packaged products of major Australian brands	Standard brand price collection: prices collected of non-packaged items and packaged products of major Australian brands ***‘Cheapest options’ price collection: prices collected of non-packaged items and packaged products of cheapest equivalent of standard brand product (including ‘own brands’)***
Household income calculation methods	Median gross household income of area sampled Low-minimum disposable household income	Low-minimum disposable household income ***Welfare dependent household income***
Analysis and reporting methods	Cost and affordability of habitual and recommended diets reported	Cost and affordability of habitual and recommended diets reported for both ‘standard brand’ price collection ***and ‘cheapest option’ price collection***.

** Key changes from the Original Healthy Diets ASAP Protocol are highlighted in ***bold italics***.

**Table 2:** Composition of original Healthy Diets ASAP habitual diet pricing tool for mean Australian population and Low SEG Healthy Diets ASAP habitual diet pricing tool, and recommended diet pricing tool, for Household A (two adult, two children).

Food Item	Habitual Diet (g/Fortnight)		Recommended Diet (g/Fortnight)
	Original Healthy Diets ASAP	Low SEG Healthy Diets ASAP	Original Healthy Diets ASAP
**Energy (kJ/day)**	33,602 kJ	32,517 kJ	32,996 kJ
**Water**			
Water, bottled (mL)	5296	3485 (34% < Original)	5296
**Fruit**			
Apples (g)	3497	3638	5460
Bananas (g)	899	795	5460
Oranges (g)	1664	971	5460
Fruit salad, canned in juice (g)	2046	1544	0
Total Fruit (g)	11,133	9614 (14% < Original)	16,380
**Vegetables and Legumes**			
Potato, loose (g)	1460	1844	2320
Broccoli, loose (g)	422	389	1470
White cabbage, loose (g)	235	175	1470
Iceberg lettuce, whole (g)	795	704	1470
Carrot, loose (g)	753	618	2205
Pumpkin (g)	240	330	2205
Onion, loose (g)	84	106	1638
Tomatoes, loose (g)	488	654	1638
Sweetcorn, canned (g)	206	216	1160
Four bean mix, canned (g)	74	61	1005
Diced tomatoes, canned (g)	235	175	1638
Baked Beans, canned (g)	369	237	1005
Frozen mixed vegetables (g)	1184	746	1638
Frozen peas (g)	273	334	1638
Total Vegetables and Legumes (g)	7584	7136 (6% < Original)	22,500
**Grain (Cereal) Foods—Wholegrain and Refined**			
Wholemeal bread, pre-packaged (g)	1054	870	4272
White bread, pre-packaged (g)	3033	3001	893
Rolled oats (g)	870	578	6648
Breakfast cereal, corn flakes (g)	680	509	670
Breakfast cereal, wheat biscuits (g)	430	243	2216
White pasta (g)	1326	988	2042
White rice (g)	1622	1904	2042
Dry wheat crackers, water crackers (g)	258	89	781
Total Grain (Cereal) Foods (g)	9393	8336 (11% < Original)	19,564
**Lean Meats and Poultry, Fish, Eggs, Nuts and Seeds**			
Tuna, canned in oil (g)	1052	760	1841
Beef mince, lean (g)	267	163	1168
Lamb loin chops (g)	257	333	1169
Beef rump steak (g)	1056	1042	1172
Eggs (g)	872	884	2208
Chicken, cooked whole (g)	1661	1093	1471
Peanuts, roasted, unsalted (g)	0	0	780
Total Lean Meats and Poultry, Fish, Eggs, Nuts and Seeds (g)	5931	4822 (19% < Original)	9809
**Milk, Yoghurt, Cheese and Alternatives**			
Cheddar cheese, full fat (g)	624	682	704
Cheddar cheese, reduced fat (g)	44	49	516
Milk, full fat (mL)	5961	7301	6438
Milk, reduced fat (mL)	2929	1839	12,000
Flavoured milk (mL)	2416	2187	0
Yoghurt, full fat, plain (g)	204	101	2576
Yoghurt, flavoured reduced fat (g)	676	722	5100
Total Milk, Yoghurt, Cheese and Alternatives (g)	12,854	12,881 (0.2% > Original)	27,334
**Unsaturated Oils and Spreads (or foods from which these are derived)**			
Sunflower oil (mL)	7	15	291
Olive oil (mL)	7	15	291
Canola margarine (g)	170	197	412
Total Unsaturated Oils and Spreads (g)	184	227 (23% > Original)	994
**Discretionary Choices—other**			
Chicken soup, canned (g)	1340	2219	0
Muffin, commercial (g)	1455	922	0
Instant noodles, wheat based (g)	381	227	0
White sugar (g)	566	714	0
Cream-filled sweet biscuit, pre-packaged (g)	496	628	0
Muesli bar, pre-packaged (g)	373	339	0
Savoury flavoured biscuits (g)	222	207	0
Nuts, mixed, salted (g)	255	262	0
Confectionary (g)	418	396	0
Chocolate (g)	441	359	0
Potato crisps, pre-packaged (g)	518	650	0
Salad dressing (g)	277	211	0
Tomato sauce (g)	569	511	0
Beef sausages (g)	1047	1036	0
Butter (g)	280	195	0
Ham (g)	189	143	0
Frozen lasagne, pre-packaged (g)	4322	3684	0
Fish fillet crumbed, pre-packaged (g)	302	433	0
Ice cream (g)	1830	1307	0
Total Discretionary Choices—other (g)	18,308	17109 (7% < Original)	0
**Alcoholic Drinks**			
Beer, full strength (mL)	4661	5060	0
White wine, sparkling (mL)	863	546	0
Whisky (mL)	266	453	0
Red wine (mL)	1078	519	0
Total Alcoholic Drinks (mL)	6868	6578 (4% < Original)	0
**Takeaway foods**			
Pizza, commercial (g)	1182	1800	0
Meat pie, commercial (g)	1638	1554	0
Hamburger, commercial (g)	2413	2710	0
Potato chips, commercial (g)	670	833	0
Total Takeaway Foods (g)	5903	6897 (17% > Original)	0
**Sugar sweetened beverages**			
Sugar-sweetened soft drink (mL)	12,012	16,288 (36% > Original)	0
**Artificially sweetened drinks**			
Artificially sweetened soft drink (mL)	2390	1406 (41% < Original)	0
**Items allocated to more than one food group**			
Sandwich, pre-made, white bread, chicken, and salad * (g)	361	462	360
Canned meat and vegetable casserole ** (g)	1291	786	0
Orange fruit juice *** (mL)	6053	5331	0

* Divided equally between Grains etc, Lean meats etc, and Vegetables; ** Divided equally between Lean meats etc and Vegetables; *** Divided equally between Fruit and Discretionary choices—other.

## Data Availability

The data presented in this study are available in this article and its Supplementary Materials.

## Supplementary Materials

The following are available online at https://www.mdpi.com/article/10.3390/nu13082900/s1, Table S1: Participant numbers in subcategories in the ABS National Nutrition and Physical Activity Survey; Table S2: Concordance between ABS NNPAS food codes and food and drink items of the Healthy Diets ASAP habitual diet pricing tool; Table S3: Calculations for minimum wage and welfare only household incomes for 2019; Table S4A: Composition of original Healthy Diets ASAP habitual diet pricing tool for mean Australian population and, Low SEG Healthy Diets ASAP habitual diet pricing tool, and recommended diet pricing tool, for Household B (one adult, two children); Table S4B: Composition of original Healthy Diets ASAP habitual diet pricing tool for mean Australian population and Low SEG Healthy Diets ASAP habitual diet pricing tool, and recommended diet pricing tool for Household C (older retired couple); Table S5: Assumptions and resultant incomes for minimum wage and welfare only incomes for reference Households A, B and C in 2019; Table S6A: Dietary costs and affordability by food group and food group components of low SEG habitual and recommended diets, using ‘standard brand’ and ‘cheapest option’ prices, and comparison to the mean Australian population, for reference Household A: Two adults, two children; Table S6B: Dietary costs and affordability by food group and food group components of low SEG habitual and recommended diets, using ‘standard brand’ and ‘cheapest option’ prices, and comparison to the mean Australian population, for reference Household B: One adult, two children; Table S6C: Dietary costs and affordability by food group and food group components of low SEG habitual and recommended diets, using ‘standard brand’ and ‘cheapest option’ prices, and comparison to the mean Australian population, for reference Household C: Older retired couple.
