# Supplementary material for: Cost and Affordability of Healthy, Equitable and Sustainable Diets in Low Socioeconomic Groups in Australia
Source: Nutrients. 2021 Aug 23;13(8):2900. doi: 10.3390/nu13082900 (PMC8402089; doi:10.3390/nu13082900)
Supplement: Supplementary file 1 [file nutrients-13-02900-s001.zip › nutrients-1328223-supplementary--/Supplementary files/Bespoke tool paper Supp Table 1 Sample numbers in AHS NNPAS.pdf]

Supplementary Table S1: Participant numbers in subcategories in the ABS National Nutrition and Physical Activity Survey

| | **All Income levels** | **Lowest Income Quintile** |
|---|---|---|
| **Male 31-50, (n)** | 1669 | 151 |
| **Female 31-50, (n)** | 1896 | 258 |
| **Male 71+, (n)** | 533 | 215 |
| **Female 71+, (n)** | 745 | 338 |
| **Boy 14, (n)** | 72 | 10 |
| **Boy 14-18, (n)** | 403 | 49 |
| **Girl 8, (n)** | 67 | 9 |
| **Child 4-8, (n)** | 789 | 120 |
