# Supplementary material for: Cost and Affordability of Healthy, Equitable and Sustainable Diets in Low Socioeconomic Groups in Australia
Source: Nutrients. 2021 Aug 23;13(8):2900. doi: 10.3390/nu13082900 (PMC8402089; doi:10.3390/nu13082900)
Supplement: Supplementary file 1 [file nutrients-13-02900-s001.zip › nutrients-1328223-supplementary--/Supplementary files/Bespoke tool paper Supp Table 3 Income calculations.pdf]

Supplementary Table S3: Calculations for minimum wage and welfare only household incomes for 2019.

Household	Household A – adult male, adult female, teen boy, child		Household B - adult female, teen boy, child		Household C – Senior male, senior female
Income source	Minimum wage	Welfare only	Minimum wage	Welfare only	Welfare only
Paid employment - adult male	$1481.24	n/a	n/a	n/a	n/a
Paid employment - adult female	$233.88	n/a	$877.13	n/a	n/a
Unemployment Allowance – adult male	n/a	$504.70	n/a	n/a	n/a
Unemployment Allowance – adult female	n/a	$504.70	$302.80	$604.70	n/a
Family Tax Benefit A fortnightly payment	$428.40	$428.40	$428.40	$428.40	n/a
Family Tax Benefit A annual supplement	$57.84 ($751.90 per child per year)	$57.84 ($751.90 per child per year)	$57.84 ($751.90 per child per year)	$57.84 ($751.90 per child per year)	n/a
Family Tax Benefit B fortnightly payment	$107.66	$52.08	$110.60	$110.60	n/a
Family Tax Benefit B annual supplement	$14.04 ($365 per family per year)	$14.04 ($365 per family per year)	$14.04 ($365 per family per year)	$14.04 ($365 per family per year)	n/a
Age Pension fortnightly payment	n/a	n/a	n/a	n/a	$1271.80
Age Pension Fortnightly Supplement	n/a	n/a	n/a	n/a	$103.20
Total Clean Energy Supplement	-	$15.80	$9.50	$9.50	$21.20
Rent Assistance	$161.14	$162.12	$161.14	$162.12	$129.20
Income tax payable (including tax offsets & Medicare levy if applicable)	-$125.87	n/a	-$64.26	n/a	n/a
**FORTNIGHTLY INCOME TOTAL**	**$2358.33**	**$1739.68**	**$1897.18**	**$1387.20**	**$1525.40**
