# Supplementary material for: Cost and Affordability of Healthy, Equitable and Sustainable Diets in Low Socioeconomic Groups in Australia
Source: Nutrients. 2021 Aug 23;13(8):2900. doi: 10.3390/nu13082900 (PMC8402089; doi:10.3390/nu13082900)
Supplement: Supplementary file 1 [file nutrients-13-02900-s001.zip › nutrients-1328223-supplementary--/Supplementary files/Bespoke tool paper Supp Table 4 Diet compositions for HHB and HHC.pdf]

Supplementary Table S4A: Composition of original Healthy Diets ASAP habitual diet pricing tool for mean Australian population and, Low SEG Healthy Diets ASAP habitual diet pricing tool, and recommended diet pricing tool, for Household B (one adult, two children)

Food item	Habitual Diet (g/fortnight)		Recommended Diet (g/fortnight) Original Healthy Diets ASAP
	Original Healthy Diets ASAP	Low SEG Healthy Diets ASAP	
**Energy (kJ/day)**	23,890 kJ	23,424 kJ	23,658 kJ
**Water**			
Water, bottled (mL)	3275	2273 (31% less than Original)	3275
**Fruit**			
Apples (g)	2774	2808	4060
Bananas (g)	606	614	4060
Oranges (g)	1304	560	4060
Fruit salad, canned in juice (g)	1425	1089	0
Total Fruit (g)	8476	7298 (14% less than Original)	12180
**Vegetables and Legumes**			
Potato, loose (g)	944	955	1620
Broccoli, loose (g)	277	209	1120
White cabbage, loose (g)	141	137	1120
Iceberg lettuce, whole (g)	468	378	1120
Carrot, loose (g)	583	485	1680
Pumpkin (g)	154	289	1680
Onion, loose (g)	37	82	1218
Tomatoes, loose (g)	301	397	1218
Sweetcorn, canned (g)	130	179	810
Four bean mix, canned (g)	50	42	480
Diced tomatoes, canned (g)	141	110	1218
Baked Beans, canned (g)	241	96	480
Frozen mixed vegetables (g)	760	482	1218
Frozen peas (g)	167	128	1218
Total Vegetables and Legumes (g)	4884	4269 (13% less than Original)	16200
**Grain (Cereal) Foods – Wholegrain and Refined**			
Wholemeal bread, pre-packaged (g)	683	607	3152
White bread, pre-packaged (g)	2344	2177	669
Rolled oats (g)	615	559	4968
Breakfast cereal, corn flakes (g)	525	289	502
Breakfast cereal, wheat biscuits (g)	263	160	1656
White pasta (g)	942	580	1499
White rice (g)	1098	1248	1499
Dry wheat crackers, water crackers (g)	225	70	585
Total Grain (Cereal) Foods (g)	6753	5837 (14% less than Original)	14530
**Lean Meats and Poultry, Fish, Eggs, Nuts and Seeds**			
Tuna, canned in oil (g)	756	553	1374
Beef mince, lean (g)	157	78	865
Lamb loin chops (g)	83	113	866
Beef rump steak (g)	683	692	868
Eggs (g)	565	524	1648
Chicken, cooked whole (g)	1004	829	1098
Peanuts, roasted, unsalted (g)	0	0	360
Total Lean Meats and Poultry, Fish, Eggs, Nuts and Seeds (g)	3738	3089 (17% less than Original)	7079
**Milk, Yoghurt, Cheese and Alternatives**			
Cheddar cheese, full fat (g)	467	509	544
Cheddar cheese, reduced fat (g)	29	39	396
Milk, full fat (mL)	4702	5328	4938
Milk, reduced fat (mL)	2102	1301	9000
Flavoured milk (mL)	1529	1375	0
Yoghurt, full fat, plain (g)	127	69	1976
Yoghurt, flavoured reduced fat (g)	467	597	3900
Total Milk, Yoghurt, Cheese and Alternatives (g)	9423	9218 (2% less than Original)	20754
**Unsaturated Oils and Spreads (or foods from which these are derived)**			
Sunflower oil (mL)	4	7	160
Olive oil (mL)	4	7	160
Canola margarine (g)	127	149	226
Total Unsaturated Oils and Spreads (g)	135	163 (21% more than Original)	546
**Discretionary Choices – other**			
Chicken soup, canned (g)	715	876	0
Muffin, commercial (g)	1104	666	0
Instant noodles, wheat based (g)	301	196	0
White sugar (g)	373	430	0
Cream-filled sweet biscuit, pre-packaged (g)	359	441	0
Muesli bar, pre-packaged (g)	301	315	0
Savoury flavoured crackers (g)	182	199	0
Nuts, mixed, salted (g)	154	197	0
Confectionary (g)	373	365	0
Chocolate (g)	333	280	0
Potato crisps, pre-packaged (g)	420	608	0
Salad dressing (g)	187	161	0

Food item	Habitual Diet (g/fortnight)		Recommended Diet (g/fortnight) Original Healthy Diets ASAP
	Original Healthy Diets ASAP	Low SEG Healthy Diets ASAP	
Tomato sauce (g)	397	375	0
Beef sausages (g)	701	670	0
Butter (g)	224	109	0
Ham (g)	129	110	0
Frozen lasagne, pre-packaged (g)	3298	2828	0
Fish fillet crumbed, pre-packaged (g)	182	247	0
Ice cream (g)	1561	1077	0
Total Discretionary Choices – other (g)	13661	12377 (9% less than Original)	0
**Alcoholic Drinks**			
Beer, full strength (mL)	428	545	0
White wine, sparkling (mL)	572	433	0
Whisky (mL)	97	371	0
Red wine (mL)	491	218	0
Total Alcoholic Drinks (mL)	1588	1567 (1% less than Original)	0
**Takeaway foods**			
Pizza, commercial (g)	872	1546	0
Meat pie, commercial (g)	1194	1099	0
Hamburger, commercial (g)	1749	1896	0
Potato chips, commercial (g)	446	631	0
Total Takeaway foods (g)	4261	5172 (21% more than Original)	0
**Sugar sweetened drinks**			
Sugar-sweetened soft drink (mL)	8612	13598 (58% more than Original)	0
**Artificially sweetened drinks**			
Artificially sweetened soft drink (mL)	1419	561 (60% less than Original)	0
**Items allocated to more than one food group**			
Sandwich, pre-made, white bread, chicken and salad* (g)	175	442	360
Canned meat and vegetable casserole** (g)	864	305	0
Orange fruit juice*** (mL)	4734	4453	0

*Divided equally between Grains etc, Lean meats etc, and Vegetables

**Divided equally between Lean meats etc and Vegetables

***Divided equally between Fruit and Discretionary choices - other

Supplementary Table S4B: Composition of original Healthy Diets ASAP habitual diet pricing tool for mean Australian population and Low SEG Healthy Diets ASAP habitual diet pricing tool, and recommended diet pricing tool for Household C (older retired couple)

Food item	Habitual Diet (g/fortnight)		Recommended Diet (g/fortnight) Original Healthy Diets ASAP
	Original Healthy Diets ASAP	Low SEG Healthy Diets ASAP	
**Energy (kJ/day)**	14,536 kJ	14,329 kJ	15,095 kJ
**Water**			
Water, bottled (mL)	923	471 (49% less than Original)	923
**Fruit**			
Apples (g)	1271	1027	2800
Bananas (g)	743	680	2800
Oranges (g)	791	645	2800
Fruit salad, canned in juice (g)	2017	1980	0
Total Fruit (g)	5774	5305 (8% less then Original)	8400
**Vegetables and Legumes**			
Potato, loose (g)	1304	1280	800
Broccoli, loose (g)	249	267	700
White cabbage, loose (g)	174	125	700
Iceberg lettuce, whole (g)	418	353	700
Carrot, loose (g)	493	442	1050
Pumpkin (g)	287	287	1050
Onion, loose (g)	128	141	840
Tomatoes, loose (g)	423	461	840
Sweetcorn, canned (g)	161	151	400
Four bean mix, canned (g)	36	13	375
Diced tomatoes, canned (g)	89	98	840
Baked Beans, canned (g)	243	129	375
Frozen mixed vegetables (g)	656	686	840
Frozen peas (g)	234	232	840
Total Vegetables and Legumes (g)	5234	5013 (4% less than Original)	10350
**Grain (Cereal) Foods – Wholegrain and Refined**			
Wholemeal bread, pre-packaged (g)	1055	1057	1280
White bread, pre-packaged (g)	1093	1267	256
Rolled oats (g)	931	1169	2160
Breakfast cereal, corn flakes (g)	331	307	192
Breakfast cereal, wheat biscuits (g)	285	201	720
White pasta (g)	351	410	480
White rice (g)	335	425	480
Dry wheat crackers, water crackers (g)	62	60	224
Total Grain (Cereal) Foods (g)	4471	4925 (10% more than Original)	5792
**Lean Meats and Poultry, Fish, Eggs, Nuts and Seeds**			
Tuna, canned in oil (g)	542	494	934
Beef mince, lean (g)	173	134	433
Lamb loin chops (g)	262	254	433
Beef rump steak (g)	547	443	434
Eggs (g)	515	491	1120
Chicken, cooked whole (g)	801	818	746
Peanuts, roasted, unsalted (g)	0	0	420
Total Lean Meats and Poultry, Fish, Eggs, Nuts and Seeds (g)	3179	2982 (6% less than Original)	4520
**Milk, Yoghurt, Cheese and Alternatives**			
Cheddar cheese, full fat (g)	250	261	520
Cheddar cheese, reduced fat (g)	25	26	363
Milk, full fat (mL)	2027	1976	4532
Milk, reduced fat (mL)	2427	2071	9126
Flavoured milk (mL)	597	482	0
Yoghurt, full fat, plain (g)	109	114	1813
Yoghurt, flavoured reduced fat (g)	373	350	3650
Total Milk, Yoghurt, Cheese and Alternatives (g)	5808	5280 (9% less than Original)	20004
**Unsaturated Oils and Spreads (or foods from which these are derived)**			
Sunflower oil (mL)	5	3	132
Olive oil (mL)	5	3	132
Canola margarine (g)	135	140	186
Total Unsaturated Oils and Spreads (g)	145	146 (1% more than Original)	450
**Discretionary Choices – other**			
Chicken soup, canned (g)	1789	1794	0
Muffin, commercial (g)	793	741	0
Instant noodles, wheat based (g)	65	90	0
White sugar (g)	491	604	0
Cream-filled sweet biscuit, pre-packaged (g)	410	393	0
Muesli bar, pre-packaged (g)	46	40	0
Savoury flavoured crackers (g)	58	47	0
Nuts, mixed, salted (g)	129	127	0
Confectionary (g)	123	124	0
Chocolate (g)	109	126	0
Potato crisps, pre-packaged (g)	52	68	0
Salad dressing (g)	101	71	0

Food item	Habitual Diet (g/fortnight)		Recommended Diet (g/fortnight) Original Healthy Diets ASAP
	Original Healthy Diets ASAP	Low SEG Healthy Diets ASAP	
Tomato sauce (g)	216	232	0
Beef sausages (g)	545	521	0
Butter (g)	188	157	0
Ham (g)	103	89	0
Frozen lasagne, pre-packaged (g)	787	875	0
Fish fillet crumbed, pre-packaged (g)	252	264	0
Ice cream (g)	825	852	0
Total Discretionary – other (g)	8034	8188 (2% more than Original)	0
**Alcoholic Drinks**			
Beer, full strength (mL)	2262	2314	0
White wine, sparkling (mL)	907	780	0
Whisky (mL)	166	200	0
Red wine (mL)	968	674	0
Total Alcoholic Drinks (mL)	4303	3968 (8% less than Original)	0
**Takeaway foods**			
Pizza, commercial (g)	169	71	0
Meat pie, commercial (g)	439	464	0
Hamburger, commercial (g)	352	434	0
Potato chips, commercial (g)	134	184	0
Total Takeaway foods (g)	1094	1153 (5% more than Original)	0
**Sugar sweetened drinks**			
Sugar-sweetened soft drink (mL)	1535	1501 (2% less than Original)	0
**Artificially sweetened drinks**			
Artificially sweetened soft drink (mL)	523	382 (27% less than Original)	0
**Items allocated to more than one food group**			
Sandwich, pre-made, white bread, chicken and salad* (g)	84	86	320
Canned meat and vegetable casserole** (g)	623	639	0
Orange fruit juice*** (mL)	1903	1945	0

*Divided equally between Grains etc, Lean meats etc, and Vegetables

**Divided equally between Lean meats etc and Vegetables

***Divided equally between Fruit and Discretionary choices - other
