# Supplementary material for: Cost and Affordability of Healthy, Equitable and Sustainable Diets in Low Socioeconomic Groups in Australia
Source: Nutrients. 2021 Aug 23;13(8):2900. doi: 10.3390/nu13082900 (PMC8402089; doi:10.3390/nu13082900)
Supplement: Supplementary file 1 [file nutrients-13-02900-s001.zip › nutrients-1328223-supplementary--/Supplementary files/Bespoke tool paper Supp Table 5 Assumptions for incomes.pdf]

Supplementary Table S5: Assumptions and resultant incomes for minimum wage and welfare only incomes for reference Households A, B and C in 2019

Household	Household A:<br>Adult male (31-50y), Adult female (31-50y), teenage boy (14-18y), girl (4-8y)	Household B:<br>Adult female (31-50y), teenage boy (14-18y), girl (4-8y)	Household C:<br>Senior male (71+y), Senior female (71+y)
Minimum wage assumptions	• Adult male works on a permanent basis at national minimum wage for 38 hours a week ($19.49/hr)<br>• Adult female works on a part-time basis at national minimum wage ($19.49/hr) for 6 hours a week<br>• Both children attend school<br>• None of the family are disabled<br>• No savings or investments<br>• Private rent	• Adult female works on a casual basis at national minimum wage ($23.39/hr($19.49/hr + 15% casual loading)) for 25 hours a week for 39 weeks per year (not during school holidays)<br>• No child support received<br>• Both children attend school<br>• None of the family are disabled<br>• No savings or investments<br>• Private rent	N/A
Fortnightly Income:	$2358.33	$1897.18	N/A
Welfare only assumptions	• Both adults are unemployed and looking for work<br>• Both children attend school<br>• None of the family are disabled<br>• No savings or investments<br>• Private rent	• Adult female is unemployed and looking for work<br>• No child support received<br>• Both children attend school<br>• None of the family are disabled<br>• No savings or investments<br>• Private rent	• Neither are in paid employment<br>• Both receive the full age pension (maximum rate)<br>• Neither are disabled or frail-aged<br>• The couple has no dependent children<br>• Private rent
Fortnightly Income:	$1739.68	$1387.20	$1525.40
