# Supplementary material for: Cost and Affordability of Healthy, Equitable and Sustainable Diets in Low Socioeconomic Groups in Australia
Source: Nutrients. 2021 Aug 23;13(8):2900. doi: 10.3390/nu13082900 (PMC8402089; doi:10.3390/nu13082900)
Supplement: Supplementary file 1 [file nutrients-13-02900-s001.zip › nutrients-1328223-supplementary--/Supplementary files/Bespoke tool paper Supp table 6 detailed dietary costs HHA, HHB, HHC.pdf]

Supplementary Table S6A: Dietary costs and affordability by food group and food group components of low SEG habitual and recommended diets, using ‘standard brand’ and ‘cheapest option’ prices, and comparison to the mean Australian population, for reference Household A: Two adults, two children.

Food group and food group component costs per fortnight										
	Original Healthy Diets ASAP		Low SEG - Standard brands		Recommended Diet – Standard brands		Low SEG – Cheapest options		Recommended Diet – Cheapest options	
Food group and food group component	Habitual Diet Cost (A$)	Proportion of total diet cost (%)	Habitual Diet Cost (A$)	Proportion of total diet cost (%)	Recommended Diet Cost (A$)	Proportion of total diet cost (%)	Habitual Diet Cost (A$)	Proportion of total diet cost (%)	Recommended Diet Cost (A$)	Proportion of total diet cost (%)
Water	$18.76	2%	$12.34	2%	$18.76	3%	$6.18	1%	$9.39	2%
Fruit	$53.29	7%	$45.66	6%	$72.83	12%	$40.27	8%	$68.54	16%
Veg. & Legumes	$41.59	5%	$38.83	5%	$101.36	16%	$33.77	7%	$83.42	19%
Grains & Cereals	$43.19	6%	$36.70	5%	$108.10	17%	$20.09	4%	$49.57	11%
Meats, nuts, seeds, egg	$104.00	14%	$93.34	12%	$203.88	33%	$71.07	15%	$151.86	35%
Milk, yoghurt, cheese	$49.11	6%	$49.09	7%	$113.77	18%	$34.07	7%	$67.55	16%
Unsaturated oils & spreads	$1.24	<1%	$1.56	<1%	$7.83	1%	$0.68	<1%	$3.93	1%
Artificially sweetened soft drink	$6.02	1%	$3.54	<1%	--	--	$0.99	<1%	--	--
Other Discretionary	$178.10	23%	$166.11	22%	--	--	$92.23	19%	--	--
Alcoholic drinks	$91.08	12%	$90.36	12%	--	--	$74.53	15%	--	--
Take-away foods	$145.36	19%	$172.15	23%	--	--	$96.50	20%	--	--
Sugar sweetened soft drinks	$30.27	4%	$41.05	5%	--	--	$11.51	2%	--	--
**Total cost**	**$762.01**	100%	**$750.72**	**100%**	**$626.52**	**100%**	**$481.89**	**100%**	**$434.25**	**100%**
Healthy foods and drinks	$317.20	42%	$281.06	37%	$626.52	100%	$207.12	43%	$434.25	100%
Discretionary foods and drinks	$444.81	58%	$469.67	63%	--	--	$274.77	57%	--	--

Income and Diet affordability (% of income)					
	Original Healthy Diets ASAP	Low SEG - Standard brands	Recommended Diet – Standard brands	Low SEG - Cheapest options	Recommended Diet – Cheapest options
Income category and amount (A$)	Habitual Diet affordability	Habitual Diet affordability	Recommended Diet affordability	Habitual Diet affordability	Recommended Diet affordability
Welfare only household income $1,740 per fortnight	44%	43%	36%	28%	25%
Minimum wage household income $2,358 per fortnight	32%	32%	27%	20%	18%

Supplementary Table S6B: Dietary costs and affordability by food group and food group components of low SEG habitual and recommended diets, using 'standard brand' and 'cheapest option' prices, and comparison to the mean Australian population, for reference Household B: One adult, two children.

Food group and food group component costs per fortnight										
	Original Healthy Diets ASAP		Low SEG - Standard brands		Recommended Diet – Standard brands		Low SEG - Cheapest options		Recommended Diet – Cheapest options	
Food group and food group component	Habitual Diet Cost (A$)	Proportion of total diet cost (%)	Habitual Diet Cost (A$)	Proportion of total diet cost (%)	Recommended Diet Cost (A$)	Proportion of total diet cost (%)	Habitual Diet Cost (A$)	Proportion of total diet cost (%)	Recommended Diet Cost (A$)	Proportion of total diet cost (%)
Water	$11.60	2%	$8.05	2%	$11.60	3%	$4.03	1%	$5.80	2%
Fruit	$40.18	8%	$33.99	7%	$54.15	12%	$30.09	10%	$50.96	16%
Veg. & Legumes	$25.94	5%	$24.19	5%	$73.37	16%	$21.36	7%	$61.13	19%
Grains & Cereals	$30.65	6%	$27.68	5%	$80.33	17%	$15.53	5%	$36.71	11%
Meats, nuts, seeds, egg	$64.07	13%	$58.03	11%	$149.81	33%	$42.97	14%	$110.98	35%
Milk, yoghurt, cheese	$34.97	7%	$35.40	7%	$86.91	19%	$24.21	8%	$51.59	16%
Unsaturated oils & spreads	$0.91	<1%	$1.11	<1%	$4.30	1%	$0.48	<1%	$2.16	1%
Artificially sweetened soft drink	$3.58	1%	$1.41	<1%	--	--	$0.40	<1%	--	--
Other Discretionary	$133.02	27%	$123.74	24%	--	--	$67.57	22%	--	--
Alcoholic drinks	$27.05	5%	$36.66	7%	--	--	$26.58	9%	--	--
Take-away foods	$104.40	21%	$129.14	25%	--	--	$69.51	22%	--	--
Sugar sweetened soft drinks	$21.70	4%	$34.27	7%	--	--	$9.61	3%	--	--
**Total cost**	**$498.07**	**100%**	**$513.68**	**100%**	**$460.46**	**100%**	**$312.33**	**100%**	**$319.34**	**100%**
Healthy foods and drinks	$211.89	43%	$189.88	37%	$460.46	100%	$139.06	45%	$319.34	100%
Discretionary foods and drinks	$286.17	57%	$323.80	63%	--	--	$173.26	55%	--	--

Income and Diet affordability (% of income)					
	Original Healthy Diets ASAP	Low SEG - Standard brands	Recommended Diet – Standard brands	Low SEG - Cheapest options	Recommended Diet – Cheapest options
Income category and amount (A$)	Habitual Diet affordability	Habitual Diet affordability	Recommended Diet affordability	Habitual Diet affordability	Recommended Diet affordability
Welfare only household income $1,387 per fortnight	36%	37%	33%	23%	23%
Minimum wage household income $1,897 per fortnight	26%	27%	24%	16%	17%

Supplementary Table S6C: Dietary costs and affordability by food group and food group components of low SEG habitual and recommended diets, using 'standard brand' and 'cheapest option' prices, and comparison to the mean Australian population, for reference Household C: Older retired couple.

Food group and food group component costs per fortnight										
	Original Healthy Diets ASAP		Low SEG - Standard brands		Recommended Diet – Standard brands		Low SEG - Cheapest options		Recommended Diet – Cheapest options	
Food group and food group component	Habitual Diet Cost (A$)	Proportion of total diet cost (%)	Habitual Diet Cost (A$)	Proportion of total diet cost (%)	Recommended Diet Cost (A$)	Proportion of total diet cost (%)	Habitual Diet Cost (A$)	Proportion of total diet cost (%)	Recommended Diet Cost (A$)	Proportion of total diet cost (%)
Water	$3.27	1%	$1.67	1%	$3.27	1%	$0.83	<1%	$1.61	1%
Fruit	$31.35	10%	$28.91	9%	$37.35	13%	$23.96	12%	$34.05	17%
Veg. & Legumes	$25.19	8%	$24.44	8%	$47.99	16%	$20.80	10%	$39.14	19%
Grains & Cereals	$22.06	7%	$23.71	8%	$35.01	12%	$10.99	5%	$16.60	8%
Meats, nuts, seeds, egg	$59.58	18%	$54.13	17%	$88.80	30%	$40.28	19%	$61.61	30%
Milk, yoghurt, cheese	$20.48	6%	$19.29	6%	$82.30	28%	$12.68	6%	$48.11	24%
Unsaturated oils & spreads	$0.98	<1%	$0.98	<1%	$3.55	1%	$0.41	<1%	$1.75	1%
Artificially sweetened soft drink	$1.32	<1%	$0.96	<1%	--	--	$0.27	<1%	--	--
Other Discretionary	$67.38	21%	$67.92	22%	--	--	$41.48	20%	--	--
Alcoholic drinks	$61.78	19%	$56.85	18%	--	--	$39.63	19%	--	--
Take-away foods	$26.04	8%	$28.16	9%	--	--	$15.52	7%	--	--
Sugar sweetened soft drinks	$3.87	1%	$3.78	1%	--	--	$1.06	1%	--	--
**Total cost**	**$323.30**	**100%**	**$310.79**	**100%**	**$298.27**	**100%**	**$207.92**	**100%**	**$207.79**	**100%**
Healthy foods and drinks	$164.23	51%	$154.08	50%	$298.27	100%	$110.22	53%	$207.79	100%
Discretionary foods and drinks	$159.07	49%	$156.72	50%	--	--	$97.70	47%	--	--

Income and Diet affordability (% of income)					
	Original Healthy Diets ASAP	Low SEG - Standard brands	Recommended Diet – Standard brands	Low SEG - Cheapest options	Recommended Diet – Cheapest options
Income category and amount (A$)	Habitual Diet affordability	Habitual Diet affordability	Recommended Diet affordability	Habitual Diet affordability	Recommended Diet affordability
Welfare only household income $1,525 per fortnight	21%	20%	20%	14%	13%
